# A prospective, multi-centre trial of PSMA-PET compared to FDG-PET for staging of newly diagnosed high risk prostate cancer

**DOI:** 10.1186/s13550-025-01265-z

**Published:** 2025-07-24

**Authors:** Matthew J. Roberts, Natasha A. Roberts, Anita Pelecanos, John W. Yaxley, Simon J. D. Harley, Amila R. Siriwardana, Karla Cullen, Marita Prior, Karen Lindsay, Ian Vela, Anna Kuchel, Nattakorn Dhiantravan, Paul Thomas, David A. Pattison

**Affiliations:** 1 Department of Urology, Royal Brisbane and Women’s Hospital, Brisbane, QLD 4006 Australia; 2 Faculty of Medicine, The University of Queensland, Brisbane, Australia; 3 Faculty of Medicine, UQ Centre for Clinical Research, The University of Queensland, Brisbane, QLD Australia; 4 Department of Urology, Redcliffe Hospital, Brisbane, QLD Australia; 5 STARS Education and Research Alliance, Metro North Health and The University of Queensland, Brisbane, Australia; 6 QIMR Berghofer Medical Research Institute, Brisbane, QLD Australia; 7 School of Nursing, Midwifery and Social Work, The University of Queensland, Brisbane, QLD Australia; 8 Wesley Urology Clinic, The Wesley Hospital, Brisbane, QLD Australia; 9 Herston Imaging Research Facility, The University of Queensland, Brisbane, Australia; 10 Department of Urology, Princess Alexandra Hospital, Brisbane, QLD Australia; 11 Department of Medical Oncology, Royal Brisbane and Women’s Hospital, Brisbane, QLD Australia; 12 Department of Nuclear Medicine, Royal Brisbane and Women’s Hospital, Brisbane, QLD Australia

**Keywords:** Prostate cancer, Prostate-specific membrane antigen, PSMA, PET/CT, 18F-FDG, Cancer staging

## Abstract

**Background:**

Despite being a potentially attractive alternative molecular imaging modality due to wider availability and association with lethal disease in advanced prostate cancer, the role of fluorodeoxyglucose (FDG)- positron emission tomography (PET) at initial diagnosis compared to Prostate Specific Membrane Antigen (PSMA) PET is yet to be accurately determined. The aim of this study was to evaluate the additive benefit of FDG PET to PSMA PET in patients with newly diagnosed, high risk prostate cancer.

**Results:**

A prospective trial conducted across three sites between October-2021 and January-2023 recruited 32 participants with high risk (EAU classification) prostate cancer staged with PSMA PET-CT. FDG PET-CT was acquired centrally and reported with a standardised template. Median age was 69 years, median PSA was 14 ug/L, and most had PI-RADS 5 scores (59%) and ISUP Grade Group 5 tumours (66%).

Overall, FDG-PET did not detect any additional definite/probable metastasis according to physician interpretation. All tumours showed PSMA avidity and higher stage was observed per PSMA-PET in 5 participants. No FDG uptake at the primary tumour occurred in 34% of participants. FDG-PET did not result in a change in management for any participant. PSA remission rates were lower in patients with stage ≥ 3 tumours on MRI (60% vs 94%, p = 0.04). Patient reported outcomes (PROs) were largely stable throughout the study.

**Conclusions:**

FDG-PET did not provide additive staging information above PSMA-PET or alter management for newly diagnosed high-risk prostate cancer patients.

*Trial registration number***:** ANZCTR ACTRN12621001185853. Registered 03–09-2021. Available at https://www.anzctr.org.au/Trial/Registration/TrialReview.aspx?id=382299

**Supplementary Information:**

The online version contains supplementary material available at 10.1186/s13550-025-01265-z.

## Introduction

Accurate characterisation of prostate cancer, the most common internal cancer in males, at diagnosis is important for selection of local and systemic treatments. Local treatment has the greatest impact on survival in localised (non-metastatic) [1] prostate cancer, while systemic therapy is most important for metastatic cancer [2, 3]. A key factor influencing post-treatment response is cancer staging, where conventional imaging such as computed tomography (CT) and whole-body bone scans were traditionally used despite limited diagnostic accuracy.

Molecular imaging has improved diagnostic accuracy of staging through positron emission tomography (PET), particularly in prostate cancer using prostate specific membrane antigen (PSMA) PET. The proPSMA trial showed that PSMA PET had a 27% greater accuracy than conventional imaging [4], leading to significant management impact and endorsement by international guidelines for use in prostate cancer staging [5]. While high PSMA uptake is prognostic [6, 7], PSMA-PET does not detect all prostate cancers, with 5–10% showing absent PSMA uptake [8]. These cancers may have features with worse prognosis, such as higher Gleason score [9], variant morphology (e.g. ductal, neuroendocrine) [10] and worse biochemical recurrence-free survival [7]. Thus, alternative staging strategies warrant consideration.

Among the first PET agents, use of 18F-fluorodeoxyglucose (FDG) PET for prostate cancer was limited due to disappointing early results [11] and then inferior performance compared to other PET agents (e.g. 11C-Choline) [12]. For these reasons, current Appropriate Use Criteria for PSMA PET state that FDG PET has limited applicability in prostate cancer staging [13]. However, recent advances in radioligand therapy for and use of both PSMA and FDG PET for patient selection has reinvigorated interest into the role of FDG PET when PSMA PET is used for staging. FDG PET may be most valuable in patients with metastatic castration-resistant prostate cancer (mCRPC), aggressive tumours and negative conventional imaging [14]. Additionally, FDG PET may serve as a prognostic biomarker for patients with mCRPC [15]. Despite being a potentially attractive alternative molecular imaging modality due to wider availability [16], the role of FDG PET at initial diagnosis combined with PSMA PET is yet to be accurately determined. Therefore, the aim of this trial was to evaluate the additive benefit of FDG-PET above PSMA-PET in patients with newly diagnosed, high risk prostate cancer. Secondary aims considered histological subtype, oncological outcomes and influence on patient reported outcomes. We hypothesized that FDG PET will provide additional information, including detection of occult metastases and primary tumour foci.

## Methods

### Setting

A prospective, multi-centre trial was conducted in Brisbane, Australia that enrolled patients with histologically confirmed, high risk prostate cancer according to the EAU classification system following staging with both mpMRI and PSMA PET. Specifically, we defined high risk disease as at least one of;

- Serum PSA ≥ 20 ng/ml
- Biopsy Gleason Score ≥ 4 + 4 / International Society of Urological Pathologists (ISUP) Grade Group 4–5
-  ≥ cT3 according to digital rectal examination (DRE)

Participants were excluded if they had a history of other active malignancy within the last two years (except non-melanoma skin cancer and superficial bladder cancer), received prior radiotherapy or systemic therapies (e.g. androgen deprivation therapy, chemotherapy) for prostate cancer for longer than 21 days or severe active co-morbidity (following revision of initial trial protocol that specified use of systemic therapies as an exclusion criterion) or other barriers to completing study processes. The trial received institutional ethics approval from the Royal Brisbane and Women’s Hospital (RBWH) Human Research Ethics Committee (approval number HREC/2021/QRBW/75271) and was prospectively registered with the Australian New Zealand Clinical Trials Registry (ACTRN12621001185853).

### Imaging – mpMRI and PSMA PET

Both mpMRI and PSMA PET were acquired by a variety of hospital and community imaging centres according to the local standard of care. mpMRI was acquired prior to prostate biopsy and reported according to standard protocols. Acquired images included T2-weighted, diffusion-weighted imaging and apparent diffusion coefficient with dynamic contrast enhancement, using a 3 T machine (specific brand/type varied according to imaging provider). Experienced radiologists reported images according to the Prostate Imaging-Reporting and Data System (PI-RADS) version 2. For PSMA PET, three tracers were used, [^68^ Ga]Ga-PSMA-11 [^18^F]F-DCFPyL-PSMA and [^18^F]F-PSMA-1007 according to the preference of the imaging provider. All three tracers were considered to provide equivalent diagnostic accuracy for prostate cancer staging. Imaging acquisitions for each provider have been published previously [17–19].

### Imaging – FDG PET

FDG PET was acquired at a central imaging facility (Herston Imaging Research Facility; HIRF) on a Siemens mCT Flow PET/CT scanner. FDG PET images were reported by experienced nuclear medicine physicians with reference to prior PSMA PET according to a structured form, considering the location of primary prostatic tumour/s and metastases, with Maximum Standard Uptake Value (SUVmax) values and certainty scores provided, as well as an overall conclusion according to Nuclear Medicine physician interpretation.

Both PSMA PET and FDG PET scan results were classified according to the TNM classification.

### Outcome measures

The primary outcome of the trial was FDG PET/CT diagnostic accuracy compared to PSMA PET/CT at 12 months follow-up, based on hard and soft criteria similar to that used in the proPSMA trial (Supplementary Table 1). PSMA PET/CT was considered as the standard of reference based on published superiority to any other staging modality and recommended standard of care for staging purposes by international guidelines. For diagnostic accuracy, equivocal lesions were considered negative for metastatic disease. Secondary outcomes included management change, oncological outcomes and FDG PET result. Oncological outcomes included PSA remission after initial treatment (known to be associated with cancer-specific mortality), defined as PSA < 0.1 ug/L after radical prostatectomy [20], or PSA < 0.5 ug/L after neoadjuvant ADT prior to radiotherapy [21] [22].

Patient reported outcomes after FDG PET was assessed using the European Organisation for Research and Treatment of Cancer (EORTC) Quality of Life Questionnaire for cancer patients (QLQ‐C30) [23] at baseline and at 1, 6 and 12 months after FDG PET. Additionally, a shortened version of the Study Participant Feedback Questionnaire (SPFQ) [24] was applied.

This study was designed to initially be a pilot study of 30 participants. Recruitment occurred for 15 months for pragmatic reasons (to end of clinical year 2022).

### Statistical analysis

Characteristics were summarised with median (interquartile range) for continuous non-normally distributed variables and numbers (percent) for categorical variables. Associations between categorical variables were explored using Chi-square or Fisher’s exact test (for greater than 20% of expected cell counts less than 5). Mann–Whitney U tests were used for associations between binary and continuous non-normally distributed variables. Spearman rank correlation was used to assess correlations between two continuous variables. Statistical significance was set at a *p* value less than 0.05 (two-sided). ROC curve analysis was used to identify if SUVmax was a good classifier of PSA remission and determine a cut-point that maximised sensitivity and specificity. These analyses were performed in STATA version 15 (StataCorp., 2017, Stata Statistical Software: Release 15; College Station, TX, USA: Statacorp LLC).

## Results

### Patient characteristics

Overall, 82 potentially eligible patients were screened, 33 participants were recruited and 32 participants completed initial study processes between October-2021 and January-2023 (Supplementary Fig. 1). One participant withdrew due to logistical challenges. Participant characteristics are outlined in Table 1. In general, participants had high PSA density (median 0.35 ng/ml/cc), PI-RADS 5 score (59%) and ISUP Grade Group 5 tumours (66%). The most commonly used PSMA tracers were [^18^F]F-PSMA-1007 (44%) and [^68^ Ga]Ga-PSMA-11 (41%). The median (IQR) time interval between PSMA and FDG PET was 40 (25–56) days.

**Table 1:** Participant characteristics

Characteristic	Summary N = 32
Age, median (IQR)	69 (66–72.5)
Most recent PSA (ng/mL), median (IQR)	14 (7.5–28.5)
*Family history of prostate cancer, n (%)*	
Yes	7 (28)
No	18 (72)
PSA density, median (IQR)	0.35 (0.18–0.76)
*Multiparametric MRI highest PI-RADS score, n (%)*	
≤ 4	13 (41)
5	19 (59)
*Multiparametric MRI T stage, n (%)*	
≤ 2	16 (50)
T3a/b	16 (50)
*ISUP grade group, n (%)*	
≤ 4	11 (34)
5	21 (66)
*PSMA tracer, n (%)*	
[^18^F]F-PSMA-1007	14 (44)
[^68^ Ga]Ga-PSMA-11	13 (41)
[^18^F]F-DCFPyl	5 (16)
SUVmax [^18^F]F-PSMA-1007 (n = 14), median (IQR)	24.1 (14.4–42.4)
SUVmax [^68^ Ga]Ga-PSMA-11 (n = 13), median (IQR)	14.2 (5.6–17.3)
SUVmax [^18^F]F-DCFPyL-PSMA (n = 5), median (IQR)	13.7 (10.4–14.3)

### Staging performance

Comparison of staging performance between PSMA PET and FDG PET is presented in Table 2. Overall, FDG PET did not detect any additional definite/probable metastasis according to nuclear medicine physician interpretation. Among the 32 participants, 84% (27/32) of PET scans were concordant, primarily due to detection of localised (stage N0 M0) disease (78%; 25/32). Discordant results were due to higher stage, both regional nodal and distant metastases, detected by PSMA PET in 5 participants.

**Table 2:** Comparison of PSMA and FDG staging

PSMA stage	FDG stage			
	N0M0	N1M0	N0M1	N1M1
N0M0	25	0	0	0
N1M0	2	0	0	0
N0M1	2	0	1	0
N1M1	1	0	0	1

**Fig. 1:**
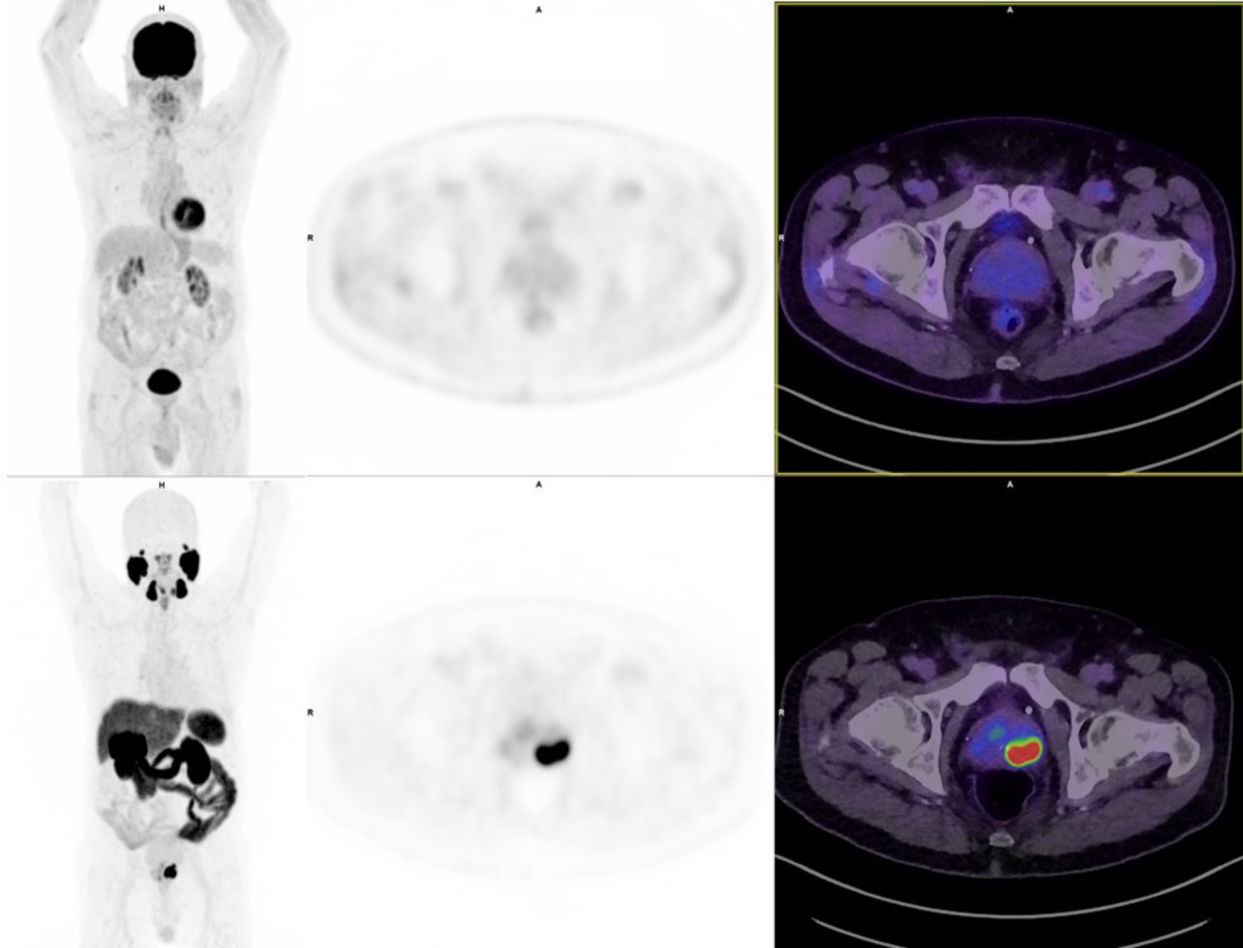
Intra-individual comparison of discordant imaging results with no uptake on [18F]FDG PET (upper row, left to right maximum intensity projection, axial PET and axial fused PET/CT, SUV window 0–10) and intense avidity on [18F]PSMA-1007 PET (lower row, SUV window 0–15) for a patient with Gleason score 4 + 5 = 9, stage pT3b prostate cancer

When the local staging of the primary prostate tumour was considered, all participants showed PSMA avidity. Three participants (9%) showed low (SUVmax < 4) PSMA avidity, of which two were ISUP 5 (PI-RADS 5 and 4) and were FDG avid (SUVmax 4.2 and 5.3). Retrospective review considering the PRIMARY score [25] (not derived at time of study commencement) revealed PRIMARY scores of 1 (n = 2) and 4 [n = 1, focal low grade (SUVmax 3.5) peripheral zone avidity). Conversely, 34% (11/32) of tumours displayed no FDG avidity, 31% (10/32) of tumours displayed low (SUVmax < 4) FDG avidity, while only 13% (4/32) showed high (SUVmax > 10) FDG avidity. Among the 5 participants with discordant PSMA PET and FDG PET results, an example shown in Fig. 1, 4 participants (80%) displayed no FDG avidity of the primary tumour.

Initiation or duration of ADT was not significantly associated with primary tumour FDG avidity (per nuclear medicine physician interpretation), nor SUVmax overall or SUVmax using ≥ 3.9 cut-off. (Supplementary Table 2).

### Management change

**Fig. 2:**
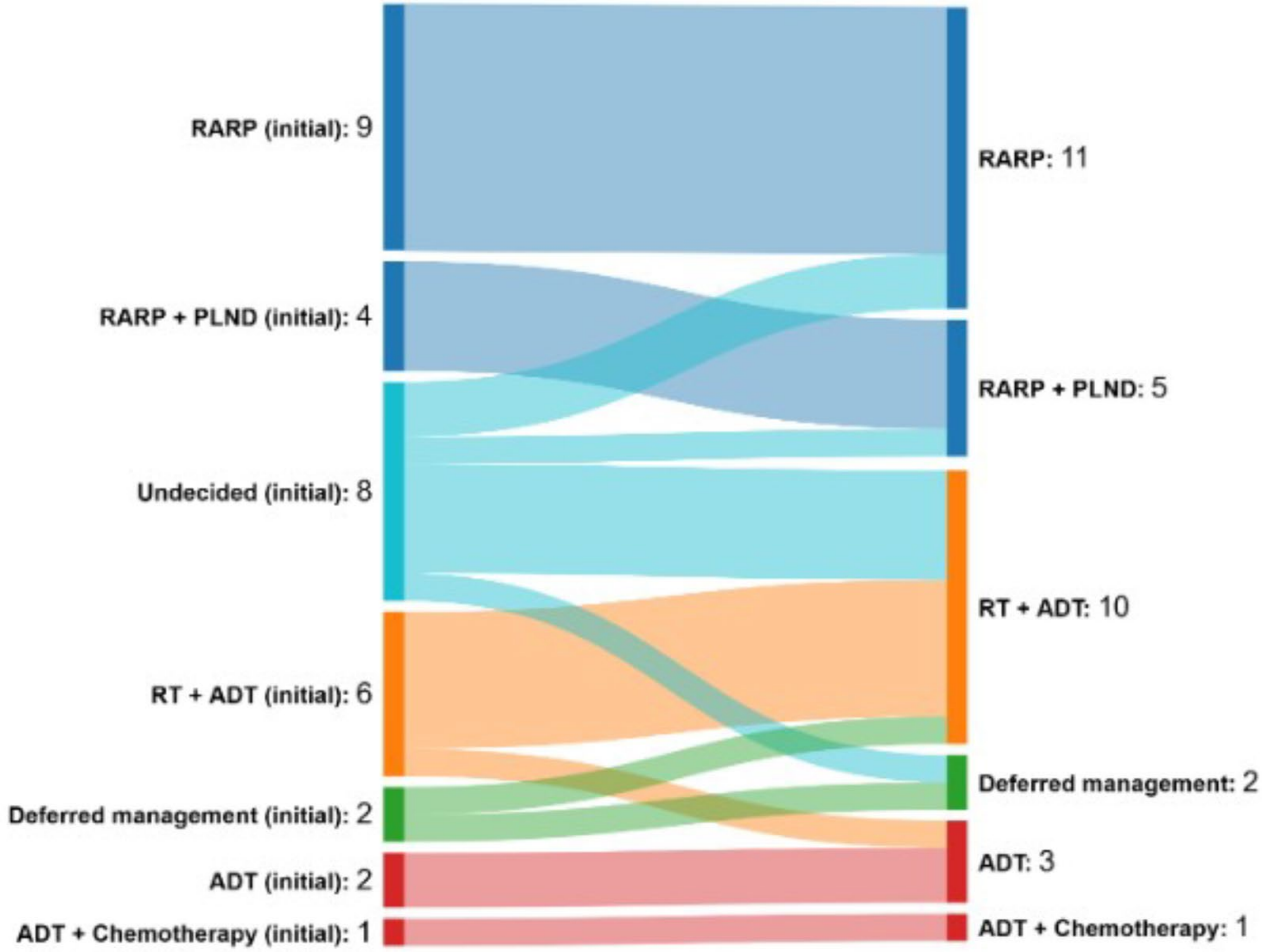
Management change from enrolment to definitive management during the trial. Abbreviations: ADT, androgen deprivation therapy; PLND, pelvic lymph node dissection; RT, radiotherapy RARP, robotic-assisted radical prostatectomy;

Change in management that occurred during the trial is outlined in Fig. 2. The most common management strategy was radical prostatectomy (16/32; 50%) and radiotherapy with ADT (10/32; 31%). No changes in management were due to FDG PET findings. The majority of participants who underwent change in management were initially undecided or within a deferred treatment pathway. Two participants received additional tests (bronchoscopy for hilar lymphadenopathy, brain MRI for FDG-avid pituitary adenoma).

### Oncological outcomes

Table 3 outlines characteristics associations with PSA remission. Participants who did not achieve PSA remission had a higher proportion of stage ≥ 3 tumours on MRI (86% vs 38%, p = 0.04) and ISUP Grade Group 5 (100% vs 58%, p = 0.07) but did not reach statistical significance (Supplementary Table 3; Supplementary Fig. 2). Participants with high FDG SUVmax of the primary tumour, defined as ≥ 3.9 based on ROC analysis (Supplementary Fig. 2), had a higher proportion of not achieving PSA remission (71% vs 29%), but was not statistically significant (p = 0.08). PSMA SUVmax of the primary tumour was not associated with PSA remission (p = 0.31).

**Table 3:** Comparison of clinical variables for PSA remission (yes vs. no) following initial treatment (surgery or hormone therapy)

Characteristic	PSA remission	No PSA remission	*p* value
	N = 24	N = 7	
Age	69 (66–73)	69 (61–75)	0.65
Most recent PSA (ng/mL)	12 (7.3–28.5)	14 (9–19)	0.79
*Family history prostate cancer*			1.00
Yes	5 (25%)	1 (25%)	
No	15 (75%)	3 (75%)	
*Multiparametric MRI highest PI-RADS score*			0.19
≤ 4	12 (50%)	1 (14%)	
5	12 (50%)	3 (86%)	
*Multiparametric MRI T stage*			0.037
≤ T2	15 (63%)	1 (14%)	
T3a/b	9 (38%)	6 (86%)	
*ISUP grade group*			0.066
≤ 4	10 (42%)	0 (0%)	
5	14 (58%)	7 (100%)	
*Intraductal or ductal component*			1.00
No	8 (62%)	3 (75%)	
Yes	5 (38%)	1 (25%)	
*PSMA tracer*			0.72
[^18^F]F-PSMA-1007	10 (42%)	4 (57%)	
[^68^ Ga]Ga-PSMA-11	10 (42%)	3 (43%)	
[^18^F]F-DCFPyL-PSMA	4 (17%)	0 (0%)	
PSMA prostate lesion SUVmax	14.4 (10.9 – 22.8)	21.8 (14.3 – 29)	0.31
*Stage per PSMA PET*			*0.30*
N0M0	20 (83%)	4 (57%)	
N1M0/N0M1/N1M1	4 (17%)	3 (43%)	
*Lesion FDG avidity*			1.00
Yes	15 (63%)	5 (71%)	
No	9 (38%)	2 (29%)	
*Lesion FDG SUVmax*			0.078
< 3.9	17 (71%)	2 (29%)	
≥ 3.9	7 (29%)	5 (71%)	

### Associations with FDG PET avidity

Table 4 shows the associations between FDG avidity of the primary tumour (per nuclear medicine physician interpretation; positive n = 21; negative n = 11) and various clinical variables. FDG positive tumours, compared to FDG negative tumours, had a higher proportion of PI-RADS score 5 lesions (71% vs 36%; p = 0.07), but this was not statistically significant. FDG negative tumours were associated with higher disease stage on PSMA PET (N1M0/N0M1/N1M1; 45% vs 10%, p = 0.03) and numerically higher median PSMA SUVmax (20.2 positive vs 14.3 negative; p = 0.15) that did not reach statistical significance. Additionally, PSMA and FDG SUVmax were not correlated (r = 0.11; p = 0.62).

**Table 4:** Comparison of clinical variables for primary tumour avidity (FDG positive vs negative)

Characteristic	FDG Positive	FDG Negative	*p* value
	N = 21	N = 11	
Age	69 (66–71)	71 (66–77)	0.33
Most recent PSA (ng/mL)	19 (7.7–30)	10 (6.3–27)	0.24
*Family history prostate cancer*			0.63
Yes	6 (33%)	1 (14%)	
No	12 (67%)	6 (86%)	
*Multiparametric MRI highest PI-RADS score*			0.072
≤ 4	6 (29%)	7 (64%)	
5	15 (71%)	4 (36%)	
*Multiparametric MRI T stage*			0.26
≤ T2	9 (43%)	7 (64%)	
T3a/b	12 (57%)	4 (36%)	
*ISUP grade group*			0.44
≤ 4	6 (29%)	5 (45%)	
5	15 (71%)	6 (55%)	
*Intraductal or ductal component*			0.32
No	6 (55%)	6 (86%)	
Yes	5 (45%)	1 (14%)	
*PSMA tracer*			0.007
[^18^F]F-PSMA-1007	5 (24%)	9 (82%)	
[^68^ Ga]Ga-PSMA-11	11 (52%)	2 (18%)	
[^18^F]F-DCFPyL-PSMA	5 (24%)	0 ( 0%)	
PSMA prostate lesion 1 SUVmax	14.3 (10.4–19.23)	20.2 (12.9–42.8)	0.15
*Stage per PSMA PET*			0.032
N0M0	19 (90%)	6 (55%)	
N1M0/N0M1/N1M1	2 (10%)	5 (45%)	

### Patient reported outcomes

Patient reported outcomes according to the EORTC QLQ‐C30 were recorded for all participants at baseline and most (23/32; 72%) at 12 months. Participants were numerically less likely to complete questionnaires if they had surgical (10/16; 63%) than non-surgical (13/16; 82%, p = 0.43) management. Overall, mean scores were stable across the 12 month period (Supplementary Table 3). Mean functional scores were high (all > 80%), indicating high functioning, while mean symptom scores mostly remained low, indicating low symptoms. Mean Fatigue scores varied between 16.7 and 23.8 (Supplementary Fig. 3A/B). Overall mean Quality of Life scores initially declined during the first 6 months, during treatment for almost all patients, then returned to baseline levels at 12 months (Supplementary Table 3, Supplementary Fig. 3C). Mean scores for participants who underwent surgical and non-surgical management were similar.

Participants were generally satisfied with their experience in the study (86% Strongly Agree or Agree) and were positive in response to all SPFQ items (86%—100%; Supplementary Table 4).

## Discussion

Molecular imaging, particularly PET, has sought to improve accuracy of staging and better inform management for improved oncological outcomes. Indeed, FDG PET is used for many malignancies and can result in incidental prostate uptake in 3% of patients, in whom 30% can result in a significant cancer diagnosis [26]. When purposely used in prostate cancer patients, FDG PET has been reported to detect additional cancer [14], and in mCRPC improve patient selection for radioligand therapy and act as a prognostic biomarker [15]. In this prospective trial of high risk, newly diagnosed prostate cancer being considered for local therapy, we found little to no value of FDG PET when PSMA PET is used. Specifically, FDG PET did not detect additional metastases, nor significantly influence management. A third of participants displayed primary tumours with no FDG avidity, with no objective associations identified aside from stage according to PSMA PET. These results are important for the following reasons.

First, this prospectively registered trial is the largest to investigate intra-individual performance of FDG PET and PSMA PET to detect metastases in high risk, newly diagnosed, hormone sensitive prostate cancer regardless of subsequent treatment. FDG PET did not detect additional metastases for any patient, resulting in no impact on management or oncological outcomes. Before this trial, limited data existed to support or refute the use of FDG PET in this clinical scenario. A pilot study comparing 18F-PSMA-1007 to FDG PET in 21 patients reported similar primary tumour (intraprostatic) detection rates (100% vs 67%, respectively) but almost twice the detection of metastatic lesions by PSMA PET [27]. Many PET lesions were benign, but further analysis of clinical and predictive variables was not performed. Also, a recently published prospective study of 42 patients with intermediate-high risk disease prior to RP did not report additive staging benefit from FDG PET [28]. Aside from these studies, minimal data exist in the hormone sensitive setting at diagnosis, mostly limited to case reports/series in patients with negative PSMA PET imaging [14]. Therefore, the current available data suggest minimal value for FDG PET compared to PSMA PET at initial diagnosis, except in uncommon circumstances of ongoing suspicion for metastatic disease and when PSMA PET is negative.

Second, different imaging phenotypes, such as patients with similar clinical characteristics (e.g. PSA, Gleason score) but different primary tumour PET avidity (positive vs negative) encourage further enquiry into underlying molecular mechanisms and subsequent clinical outcomes. Indeed, FDG-positive PSMA-negative phenotypes have worse mCRPC prognosis [29] and high FDG uptake is a prognostic biomarker in mCRPC regardless of treatment received [15]. Furthermore, these phenotypes appear to be more common in mCRPC (24–33%) than in metastatic (15%) [30], recurrent (17%) [31] or localised hormone-sensitive disease. FDG positive phenotypes have been consistently associated with prognostic factors, such as higher serum PSA and Gleason score [31], as well as ductal-dominant tumours [28]. Similar to our findings where FDG-avidity was proportionally higher in larger tumours (PIRADS 5, 79% vs. PIRADS 4, 46%) without reaching statistical significance (p = 0.07), Kim et al. also reported that tumour volume and low PSMA avidity were associated with FDG-avidity [28]. Such imaging heterogeneity is also supported by a study by Malaspina et al. [32], where 63% of bone and 46% of PSMA avid lymph nodes were concordantly FDG avid and these lesions were less affected by PSMA avidity changes invoked by ADT, potentially enabling early identification of a castrate-resistant phenotype (FDG Positive, PSMA non-responsive).

Third, glucose metabolism-related genes may contribute to proliferation and migration of primary prostate cancer cells resulting in higher FDG uptake and reduced PSMA uptake [33], including higher GLUT1 membrane expression in Ductal tumours compared to Acinar tumours [34], known to have a worse prognosis [35]. Indeed, we found that high FDG avidity (SUVmax ≥ 3.9 at the primary tumour resulted in a lower proportion of participants with PSA remission (29% vs 71%), which did not reach statistical significance (p = 0.08). Others have reported higher biochemical failure rates after RP and detection of metastases [28], clinically illustrating worse tumour biology. Similarly, reduced PSMA expression has been observed in aggressive prostate cancer variants, such neuroendocrine differentiation [36]. Low PSMA expressing tumours are uncommon, reported to be 3.3% in the proPSMA trial [4] with subsequent data that treatment failure in these patients was infrequent [37]. While genomic correlates of PSMA and FDG expression to clinical outcomes are limited, it is expected that genomic and phenotypic heterogeneity may account for variable treatment response as observed in molecular, or genetic, analyses for both localised and metastatic cancers [38].

Despite the strengths of the study, including prospective clinical trial design with inclusion of clinically relevant patients and rich clinical and imaging information, consideration of the limitations is important for translation to clinical practice. First, the study was subject to clinician discretion for referral for FDG PET and treatment planning, however the patterns observed here reflect those observed in our usual practice. Second, different PSMA PET tracers and mpMRI imaging providers were used and FDG readers were not blinded to PSMA PET results, consistent with usual practice, but less ideal for research comparisons. Third, it is acknowledged that the strength of association between SUVmax and PSA remission may be artificially inflated due to the derivation of an optimal cut-point for SUVmax by maximising the sensitivity and specificity of classifying PSA remission through the ROC curve analysis. The derived cutoff (3.9) is similar to that described by Lavallée et al. [39], where a SUVmax of ≥ 4.6 was associated with adverse surgical parameters and worse survival outcomes. This pilot study is limited by sample size and statistical power. The absence of benefit in the primary outcome meant that further expansion was not pursued.

## Conclusions

FDG-PET did not provide additive staging information above PSMA-PET or alter management for newly diagnosed high-risk prostate cancer patients. Further validation in prospective studies enriched with PSMA-negative tumours and translational studies with follow-up for oncologic outcomes is warranted.

## Supplementary Information

Supplementary file1.

## Data Availability

The datasets generated during and/or analysed during the current study are available from the corresponding author on reasonable request.
