# Supplementary material for: A prospective, multi-centre trial of PSMA-PET compared to FDG-PET for staging of newly diagnosed high risk prostate cancer
Source: EJNMMI Res. 2025 Jul 24;15:92. doi: 10.1186/s13550-025-01265-z (PMC12290150; doi:10.1186/s13550-025-01265-z)
Supplement: Supplementary file 1 — Supplementary file1. [file 13550_2025_1265_MOESM1_ESM.docx]

# **Supplementary Material**

# **Supplementary Table 1:** Diagnostic accuracy of FDG-PET compared to PSMA PET-CT at 12 months follow-up, based on composite end point based on hard and soft criteria

| **Hard criteria:** |
| --- |
| Histopathology demonstrating prostate adenocarcinoma (pathology report and/or clinical notes) |
| Change of bone lesion to sclerotic/blastic on opportunistic follow-up imaging assessment (results of imaging reports and/or clinical notes) for non-pelvic lymph node metastases |
| **Soft criteria:** |
| Typical appearance of multi-focal metastatic disease (results of imaging reports) |
| Typical appearance of a metastatic lesion on an imaging modality other than the one performed as the index scan (results of imaging reports and/or clinical notes) |
| Increase in the number or size of bone lesion(s) or soft tissue lesion(s) from one imaging exam to the next, over time following the index scan (results of imaging reports and/or clinical notes) |
| Decrease in the number or size of bone lesion(s) or soft tissue lesion(s) following disease appropriate treatment from one imaging exam to the next, over time following the index scan (results of imaging reports and/or clinical notes) |
| Presence of a lesion on an initial imaging examination with associated clinical symptoms suggesting malignancy (results of imaging reports and/or clinical notes) |
| Increasing alkaline phosphatase (ALP) or PSA in keeping with clinical scenario of progression, decreasing levels in response to treatment, or PSA >0.2 ng/ml at least three weeks following prostatectomy (laboratory results showing increase/decrease and/or clinical notes) |
| Patient received localised treatment for metastasis (e.g. radiotherapy) (clinical notes) |
| Unequivocal persistence of positive finding present on the baseline scan on repeat imaging at 6 months, in setting of PSA >0.2 ng/ml at least three weeks following prostatectomy (results of imaging reports, laboratory results and/or clinical notes) |

# **Supplementary Table 2:** Association of ADT commencement prior to FDG-PET with lesion avidity and SUVmax

|  | **ADT started** | | **p** |
| --- | --- | --- | --- |
|  | **Yes** | **No** |  |
| Lesion avidity |  |  |  |
| Yes | 9 (60%) | 12 (71%) | 0.53 |
| No | 6 (40%) | 5 (29%) |  |
| SUVmax | 3.5 (2.8 – 5.4) | 3.2 (2.5 – 4.7) | 0.69 |
| SUVmax |  |  |  |
| <3.9 | 8 (53%) | 12 (71%) | 0.31 |
| ≥3.9 | 7 (47%) | 5 (29%) |  |

# **Supplementary Table 3:** Patient reported outcomes according to the EORTC QLQ‐C30. Mean (Standard deviation) scores were calculated across domains, including Functional, Symptom and overall Quality of Life.

| **Domain** | | | **Baseline**  **N=32** | **1 month**  **N=29** | **6 months**  **N=23** | **12 months**  **N=23** |
| --- | --- | --- | --- | --- | --- | --- |
| Functional | | Physical | 92.6 (11.3) | 90.8 (11.9) | 89.0 (11.3) | 90.4 (11.7) |
|  |  | Role | 91.1 (16.4) | 84.5 (25.9) | 84.1 (21.6) | 87.0 (20.1) |
|  |  | Emotional | 82.3 (21.8) | 82.2 (21.1) | 85.9 (18.4) | 84.4 (18.7) |
|  |  | Cognitive | 89.6 (13.9) | 89.7 (16.9) | 89.1 (17.1) | 89.1 (15.6) |
|  |  | Social | 88.5 (15.5) | 88.5 (17.9) | 84.1 (19.1) | 84.8 (19.4) |
| Symptom | | Fatigue | 16.7 (13.8) | 23.8 (17.5) | 19.8 (20.1) | 21.3 (22.7) |
|  |  | Nausea and Vomiting | 4.7 (11.8) | 4.3 (9.6) | 3.3 (11.4) | 5.4 (10.5) |
|  |  | Pain | 7.8 (14.0) | 12.1 (16.6) | 16.7 (20.7) | 13.0 (24.6) |
| Quality of life | Overall | | 77.1 (19.2) | 74.1 (16.1) | 69.6 (18.2) | 76.1 (18.3) |
|  | Surgical | | 79.2 (22.2) | 76.3 (18.9) | 72.2 (24.7) | 75.0 (22.6) |
|  | Non-surgical | | 75.0 (16.1) | 72.4 (13.9) | 67.9 (13.4) | 76.9 (15.3) |

# **Supplementary Table 4:** Study Participant Feedback Questionnaire (SPFQ) Responses

|  | **Strongly Agree** | **Agree** | **Neither agree or disagree** | **Disagree** | **Strongly disagree** |
| --- | --- | --- | --- | --- | --- |
| **ENROLMENT** |  |  |  |  |  |
| 1. I understand the purpose of this study | 17 | 15 | 0 | 0 | 0 |
| 2. The information given to me before I joined the study was everything I wanted to know | 16 | 14 | 1 | 0 | 0 |
| **AFTER FDG PET** |  |  |  |  |  |
| 3. I am satisfied with the answers I have received to my questions during the study | 14 | 6 | 1 | 0 | 0 |
| 4. The time taken for this study is acceptable | 13 | 7 | 1 | 0 | 0 |
| 5. I am kept informed of my results during this study | 12 | 8 | 1 | 0 | 0 |
| **1 MONTH POST-FDG PET** |  |  |  |  |  |
| 6. Overall I am satisfied with my experience in the study | 12 | 12 | 4 | 0 | 0 |
| 7. Compared to when the study started the overall commitment was similar to what I expected. | 12 | 12 | 4 | 0 | 0 |

# **Supplementary Figure 1.** Consolidated Standards of Reporting Trials (CONSORT) flow chart for trial recruitment

**CONSORT 2010 Flow Diagram**

Assessed for eligibility (n=82)

Completed clinical follow-up (n=32)
♦

## Follow-up

## Imaging

Underwent FDG-PET (n=32)
♦

Withdrawal (n= 1; logistic reasons)

## Enrolment

Enrolled (n=33)

Excluded (n= 49)

- Prior treatment with systemic or radiotherapy (n=22)
- Declined to participate (n=17)
- Unsuitable due to medical history / comorbidities (n=6)
- Other reasons (n=4)

## Screening

# **Supplementary Figure 2.** Receiver operating characteristic curve for performance of SUVmax for the classification of PSA remission (n=31). Area under the curve 0.67 (95% confidence interval 0.44 – 0.90).

# **Supplementary Figure 3.** Mean Patient Reported Outcome scores according to the EORTC QLQ‐C30, considering Functional (A), Symptom (B) and overall Quality of Life (C) domains. Abbreviations: N&V, nausea and vomiting; QoL, quality of life.
